# An in-vitro biomechanical study of different fixation techniques for the extended trochanteric osteotomy in revision THA

**DOI:** 10.1186/1749-799X-8-7

**Published:** 2013-04-09

**Authors:** Zhonglin Zhu, Hui Ding, Hongyi Shao, Yixin Zhou, Guangzhi Wang

**Affiliations:** 1 Department of Biomedical Engineering, Tsinghua University, Beijing, China; 2 Bejing Ji Shui Tan hospital, the 4th Medical College of Peking University, Beijing, China

**Keywords:** Tension, Fixation, Biomechanics, Extended trochanteric osteotomy

## Abstract

**Background:**

The wire fixation and the cable grip fixation have been developed for the extended trochanteric osteotomy (ETO) in the revision of total hip arthroplasty (THA). Many studies reported the postoperative performance of the patients, but with little quantitative biomechanical comparison of the two fixation systems.

**Methods:**

An in-vitro testing approach was designed to record the loosening between the femoral bed and the greater trochanter after fixations. Ten cadaveric femurs were chosen in this study. Each femur underwent the THA, revision by ETO and fixations. The tension to the greater trochanter was from 0 to 500N in vertical and lateral direction, respectively. The translation and rotation of the greater trochanter with respect to the bony bed were captured by an optical tracking system.

**Results:**

In the vertical tension tests, the overall translation of the greater trochanter was observed 0.4 mm in the cable fixations and 7.0 mm in the wire fixations. In the lateral tension tests, the overall motion of the greater trochanter was 2.0 mm and 1.2° in the cable fixations, while it was 6.2 mm and 5.3° in the wire fixations. The result was significantly different between the two fixation systems.

**Conclusions:**

The stability of the proximal femur after ETO using different fixations in the revision THA was investigated. The cable grip fixation was significantly more stable than the wire fixation.

## Background

The extended trochanteric osteotomy (ETO), has proved to be an effective method in many revision total hip arthroplasty (THA) and complicated primary THA [1]. Wires and Cables were applied in the early ETO fixation [1]. Later the cable fixation system was improved to utilize a claw plate to strengthen the greater trochanter, which was called the cable grip system. The latest fixations were reported, such as using suture cord and nonmetallic cable [2,3]. ETO has many advantages, such as larger exposure during operation, lower risk of the femoral facture, and larger contact area for healing of the osteotomized bone, etc. [4-8]. Due to the failures of the fixations in biomechanical stability, the fragment was observed loosing from the femoral bed after the ETO [1,9,10].

There were several techniques to investigate the stability of the fixed proximal femur after the ETO, including clinical postoperative assessment [8,11-14], imaging studies [15-17], computational model analysis [18], and biomechanical tests [4,5,19-21]. Imaging studies has been widely used to measure the in-vivo displacement of the fragments. The limitation was that only the translations of the fragments could be measured, the forces acted to the bone were still unknown. The in-vitro biomechanical tests were able to simulate some of the in-vivo biomechanical conditions and study the motion of fragment when different loads were applied [4,5].

In this study, an in-vitro biomechanical testing approach was used to measure the stability of different fixations in the ETO. The micro-motion of the fixed fragment was captured when an increasing tension was applied to the greater trochanter. The stability of the proximal femur using different fixation techniques was compared.

## Methods

### Materials

Ten fresh-frozen cadaveric adult femurs (4 left, 6 right, 5 male, 5 female, average age 31) with similar geometric shape were chosen in this experiment which was approved by the institutional research board of the Beijing Jishuitan hospital. All specimens were anatomically normal, good in bone quality and without a history of a malignant lesion. The specimen was thawed at room temperature before testing. Each specimen was disarticulated from the acetabulum and tibia. After removal of the surrounding tissues, the femur was cut to remain 2/3 length in the proximal side [5]. The distal side was potted to a cylindrical mold filled with denture base resin (Shanghai New Century Dental Materials CO., LTD), to facilitate clamping tightly in the material testing system [5,20]. Surgical instruments for the THA and ETO were used. Implanted prosthesis was prepared for each femur (The ECHELON^◊^ Revision Hip System, Smith & Nephew). 18-gauge wires and cerclage cable (w/crimp, 1.8 mm, length 635 mm, Zimmer) were used in the fixations. A short claw plate (50.8 mm length, 22.1 mm width, Zimmer) and a long claw plate (121.4 mm length, 22.1 mm width, Zimmer), were combined with cables to help fix the resected greater trochanter. In order to maintain the constant material property of the bone, the room temperature was controlled the same and the moisturizing process was performed during the experiment.

#### Experiment preparation

A series of THA and ETO were performed to the femur by the same experienced surgeon.

**Figure 1:**
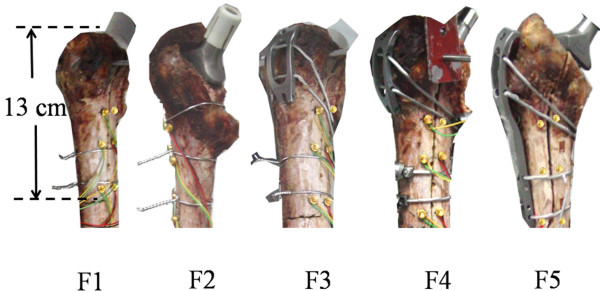
Five fixations: 2-wires fixation (F1), 3-wires fixation (F2), a short claw plate fixation with 2 wires and 2 cables (F3), a short claw plate fixation with 4 cables (F4), and a long claw plate fixation with 4 cables (F5).

**Figure 2:**
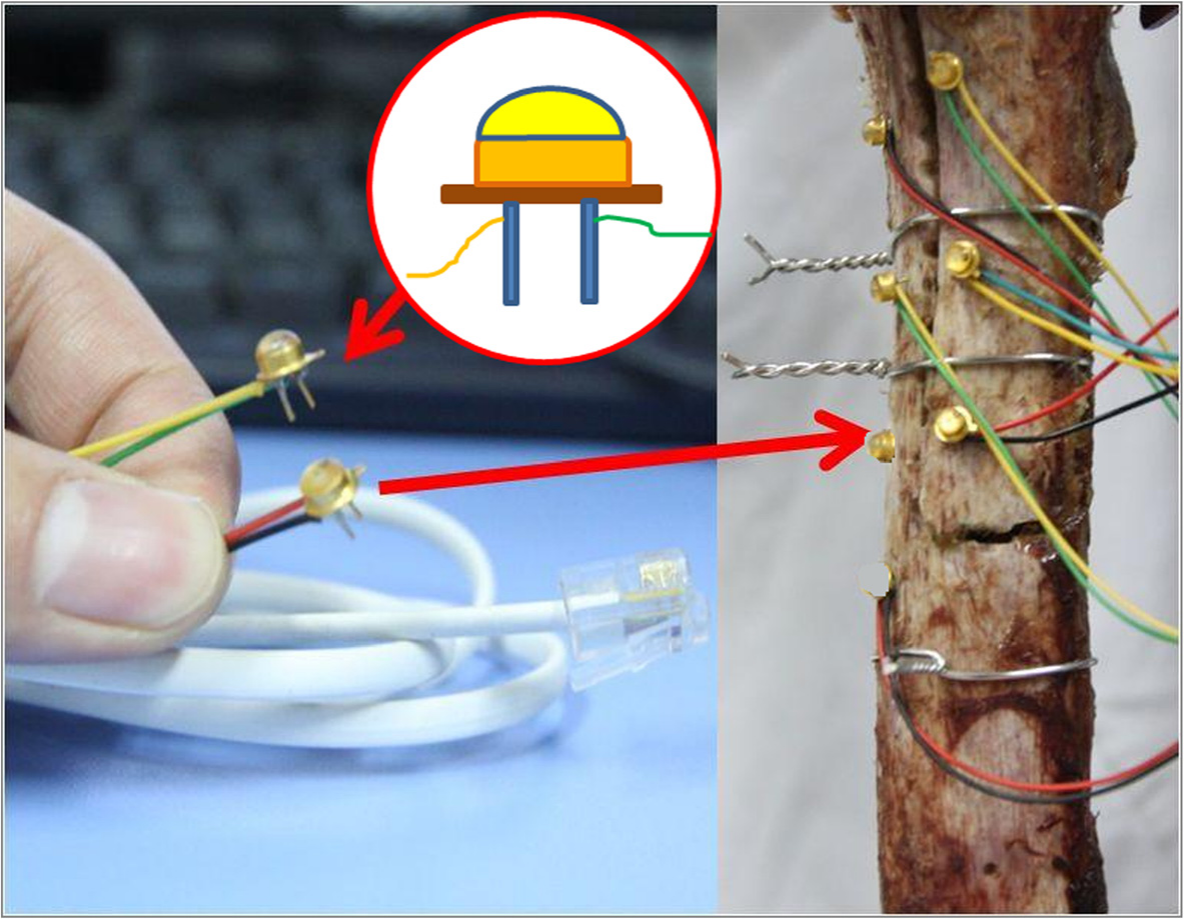
A minitype infrared active marker was used to track the 3D motion in space.

**Figure 3:**
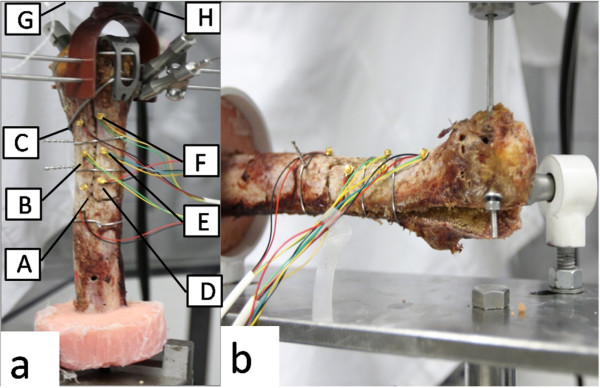
**In the vertical tension test (a), eight markers were used: the femoral bed (A, B, C), the extended greater trochanter (D, E, F), the actuator (G, H). In the lateral tension test (b), the specimen was placed horizontal.**

1) Osteotomy: The neck of the intact proximal femur was resected to implant the THA prosthesis. Then the greater trochanter was cut extendedly to remove the previous implant in a revision THA. The vertical cuts were 13 cm at the anterior and posterior side of the proximal femur (Figure 1). The horizontal cut was one third of the girth and joined both sides of the vertical cuts [7,20,22]. The piece of the extended greater trochanter was separated from the femoral bed. The THA prosthesis was replaced by an implant for revision.

2) Fixation: Each femur underwent a series of five fixations in a random order, which were 2-wires fixation (F1), 3-wires fixation (F2), a short claw plate fixation with 2 wires and 2 cables (F3), a short claw plate fixation with 4 cables (F4), and a long claw plate fixation with 4 cables (F5) (Figure 1). The short claw plate was attached to the separated bone by 2 cables. The long claw plate fixation combined 4 cables was able to fix the whole fragment. However, the short claw plate combined 2 cables was not long enough, so additional 2 wires or 2 cables were used. The wires were tightened manually by pliers. The cables were tightened semi-automatically by a synthesis tensioner with a recommended 50 kg of tension [5].

3) Measurement Devices: The three dimensional (3D) motion between the femoral bed and the fragment was tracked by an Optotrak Certus™ optoelectronic camera system (Northern Digital, Waterloo, Canada). A servo hydraulic material testing system (MTS858, with 25kN ± 5N axial force load cell, 250 Nm torque capacity, Eden Prairie, MN USA) was used to supply a tension from 0 N to 500 N with an increment of 1N per second. The sampling rate for motion data was set to 10 Hz. A self-developed minitype infrared active marker connected to the NDI system was used (Figure 2), which was validated as well as the NDI standard markers with a root-mean-square (RMS) accuracy of 0.1 mm. There were two roots on each maker to mount to the bone. Each root was less than 1 mm in diameter and 4–5 mm in length. Eight markers were used to track the motion of the femoral bed (A, B, C), the cut bone (D, E, F) and the MTS actuator (G, H) (Figure 3 (a)). The Marker D was 10 mm from the horizontal cut. The cluster of three markers on the same side was placed non-linear. The markers were placed in similar position on different specimens. Two makers that attached to the MTS actuator were used to synchronize the MTS and the motion tracking system.

#### Testing

The purpose of the test was to simulate the in-vivo mechanical environment of the proximal femur after ETO revision. The muscles attached to the greater trochanter generated a large tension during some tasks, such as lifting-up of the leg. The directions of the tensions were measured mainly in the vertical the post-lateral direction. The vertical tension on the greater trochanter guaranteed the balance of the hip joint during standing pose. The lateral tension pointed from anterior to posterior was found during standing from a seated position or climbing stairs (Figure 3 (b)). 500 N was chosen as the maximum tension in our test, similar to the previous study [5].

In the vertical tension test, the resinous cylinder was fixed on the MTS, and the greater trochanter was clamped by the vise on the actuator (Figure 3 (a)). In the lateral tension test, the implant and the resinous cylinder was fixed by a test fixture (Figure 3(b)). Before testing, a 50N tension load by MTS was performed to eliminate the gap existed in fixations and connections. After the whole fixed femur was trained to a steady state, the tension was relaxed to zero. During the test, the actuator generated a tension from 0 to 500 N with an increment of 1N per second, meanwhile the NDI system started to collect the motion data of the markers. After a pulling test the stability of the fixed femur was destroyed. The wire or cable fixations were loosened and required fixation again. The force and motion data was recorded for one kind of fixations after more than 3 trials of preconditioning biomechanical tests. The 3D motion of all makers were collected and recorded by the NDI First Principles software associated with NDI's Optotrak Certus™. The synchronous force data from 0 N to 500 N were collected by Model 793.10 Multipurpose TestWare associated with MTS.

#### Data processing

**Figure 4:**
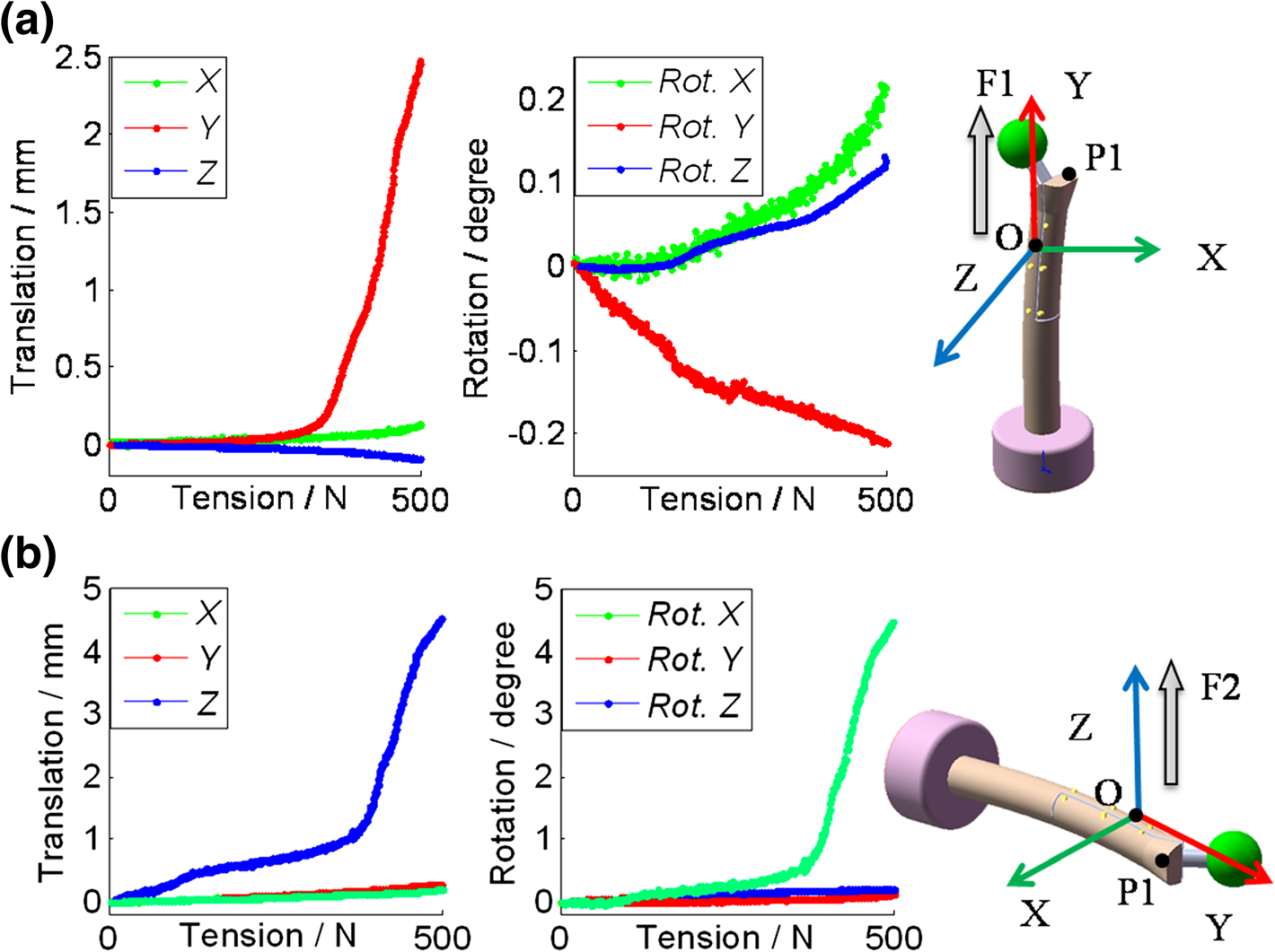
**The local coordinate system of the femoral bed: the origin was 6.5 cm from horizontal cut; The X-Y plane was decided by all the 6 markers on the bone. The 6 DOF of motion of the extended greater trochanter in one test was shown in the vertical tension test (a) and the lateral tension test (b).**

The motion of the greater trochanter relative to the femoral bed was calculated in translations and rotations {*tx,ty,tz,α,β,γ*} [23]. The deformation of each tracked object was observed less than 0.5 mm, so the femoral bed and the greater trochanter were approximately rigid bodies. During data processing, all motion data was presented in the local coordinate system (O-XYZ) of the femoral bed (Figure 4). The origin (O) of the femoral bed was 6.5 cm from the horizontal cut on the line AC (Figure 3(a)). The Y axis pointed to the marker C. The X-Y plane was decided by all the six markers (from A to F) on the bone to approximate the coronal plane using the least square method. The X axis pointed to the anterior side of the femur. The Z axis was decided using the right-handed coordinate system definition. The point P1 on the proximal end of the extended greater trochanter was 13cm from the horizontal cut on the line DF (Figure 3(a)).

In Figure 4(a), the translation along the Y axis was much larger than the translations (< 0.3 mm) in other two directions (Figure 4 (a)). Meanwhile all rotations were small (< 0.3 degree). In this study, the displacement of P1 along the Y axis was calculated to present the translations of the fragment in the vertical tension (F1) test. In Figure 4 (b), the translation along the Z axis and the rotation along the X axis were much larger than the other translations and rotations. So in the lateral tension (F2) test, the motion of the fragment was presented by the displacement of P1 along the Z axis, and the rotation was along the X axis in Figure 4(b). The motion was analyzed from 100 N to 500 N with an interval of 100 N.

The result of the five different fixations was analyzed statistically using the SPSS 16.0 software. One-way analysis of variance followed by post hoc pairwise comparisons ( Student–Newman–Keuls ) was used. Differences were considered significant at ***P<0.05***.

## Results

**Figure 5:**
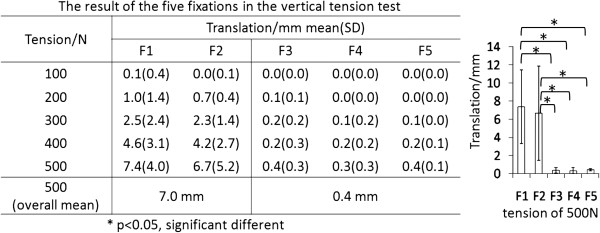
The result of the five fixations in the vertical tension test.

The motion in the vertical tension test was shown in Figure 5. All of the translations increased monotonously while the tension increased to 500 N. The statistical analysis of all fixations under the load of 500 N was given in Figure 5. The translation was 7.4 ± 4.0 mm in the test of F1 and 6.7 ± 5.2 mm in the test of F2 with no significant different between them. In the test of cable grip fixations under 500 N, the translation was 0.4 ± 0.3 mm (F3), 0.3 ± 0.3 mm (F4), and 0.4 ± 0.1 mm (F5) respectively. No significant difference was found among them. When the wire fixations (F1, F2) were compared to the cable grip system (F3, F4 and F5), a significant difference was found (***P***≦***0.001***).

**Figure 6:**
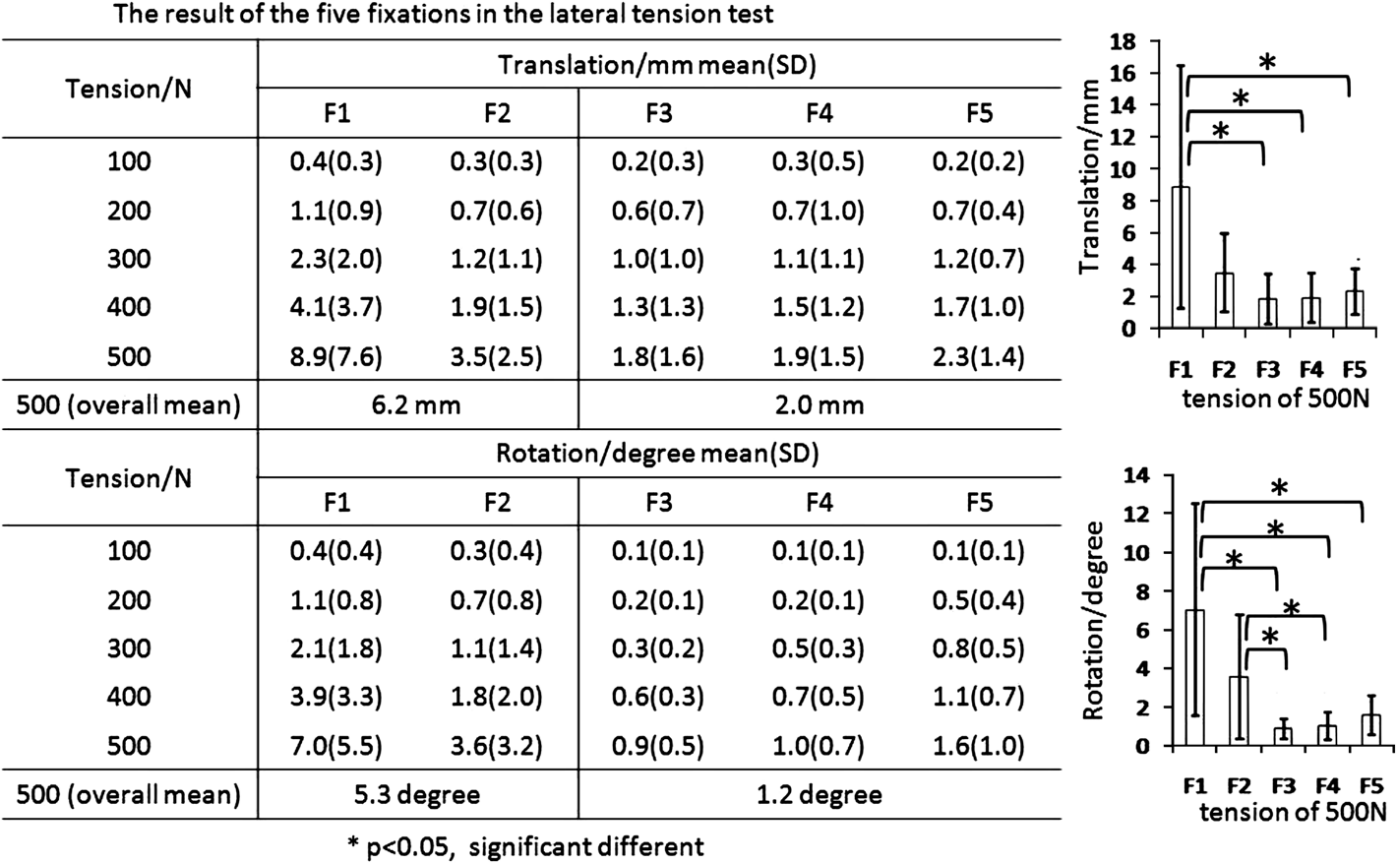
The result of the five fixations in the lateral tension test.

The result from the lateral tension test was shown in Figure 6. All of the translations and rotations increased monotonously during testing. The statistical analysis of all fixations under the load of 500 N was given in Figure 6. It was 8.9 ± 7.6 mm in translation and 7.0 ± 5.5 degree in rotation in the test of F1, and 3.5 ± 2.5 mm and 3.6 ± 3.2 degree in the test of F2, with no significant difference between them. Under the tension of 500 N, the translation was 1.8 ± 1.6 mm, 1.9 ± 1.5 mm, and 2.3 ± 1.4 mm for the F3, F4 and F5, respectively. The rotation was 0.9 ± 0.5 degree, 1.0 ± 0.7 degree, and 1.6 ± 1.0 degree for the F3, F4 and F5, respectively. No significant difference was found among them. When the wire fixations (F1) was compared with the cable grip fixations (F3, F4 and F5), a significant difference was found (***P***≦***0.015***) in both translation and rotation. There was significant difference in rotation when the test of F2 was compared to the short claw plate fixations (F3, F4).

## Discussion

The biomechanical stability of different fixations used in ETO during THA revision was investigated in this study. The stability of the cable grip fixations was better than the wire fixations.

In the vertical tension test, the experiment simulated the in-vivo abductors muscles of hip acting on the greater trochanter during standing. Kang et al. reported that 2.6 mm in average was observed after the ETO using 2-wires fixation [24]. Mardones et al. reported that the vertical translation was more than 5 mm in 7% of all patients [15]. In our study, the result was 7.4 ± 4.0 mm in the fixation using 2 wires and 6.7 ± 5.2 mm in the fixation using 3 wires. Nercessian et al. gave a standard of 2.5 mm in translation for the clinical assessment [12]. However, the average translations in the wire fixations (F1 and F2) were both larger than 2.5 mm after the tension of 300 N in this study. The maximum translation was about 0.4 mm in the claw plate fixations (F3, F4, and F5). In general, the cable grip fixations were better in stability than the wire fixations. Considering the surgical tools for fixations, all cables were tightened to 50 Kg of tension, but the wire was fixed by pliers where the tightening degree was not easy to control. This might be the reason that the standard deviation of the translation was observed larger in the wire fixations. The cables that passed the lesser trochanter were able to resist the vertical tension to provide a more stable fixation.

The translation and rotation were investigated between the femoral bed and the greater trochanter when a lateral tension was applied, which was investigated in few previous studies [20]. In this study, the rotation was observed 7.0 degree in the wire fixations under 500 N. Noble et al. observed that the stability against torsion (similar to the lateral tension) was decreased by 19% after the ETO, compared to the intact group [20]. So higher risk of nonunion or fractures might occur when lateral tension was applied to the greater trochanter. In this study, the result indicated that more wires used for fixation was able to reduce the rotation.

The result from this study was consistent with the clinical outcomes [7,13]. Shao et al. pointed out that the cable grip fixations were more stable than wire fixations. The claw plate could prevent the greater trochanter from sliding up and rotating [7]. Barrack et al. found that in the cable grip fixations the fracture of fixtures, nonunion and limping, were less than using the wire fixations [13]. In summary, the cable grip fixations were better in stability.

There were certain limitations in the current study. One limitation was that five different fixations were performed on the same femur due to the difficulty of acquiring more fresh cadavers in this study. In order to reduce the micro-damage to the cut surface, 500 N was chosen as the maximum loads. However, the micro-damage might not be ignorable, especially in a failure test such as the peak value of 970 N reported by Schwad et al. [5]. So it’s better to perform only one kind of mechanical test on one specimen. The second limitation was that the loading mode was simplified. Multiple loading modes such as loading on the femoral head would be more physiologic to simulate some biomechanical status in daily life activities, which will be used in our future study.

## Conclusions

An in-vitro biomechanical study of the stability was designed to compare the different fixations for ETO in the revision THA. The micro-motion between the fragment and the femoral bed was measured. The cable grip fixations were better in stability than the wire fixations. The comparison between the two fixation techniques was able to provide useful information to both surgeons and patients. In future studies, more specimens and multiple loading modes will be applied to test the stability of the proximal femur after revisions.

## Competing interests

The authors declare that they have no competing interests.

## Authors’ contributions

All authors were fully involved in the study and preparation of the manuscript. The manuscript has been read and agreed by all authors.
